# Fast 3D Face Reconstruction from a Single Image Using Different Deep Learning Approaches for Facial Palsy Patients

**DOI:** 10.3390/bioengineering9110619

**Published:** 2022-10-27

**Authors:** Duc-Phong Nguyen, Tan-Nhu Nguyen, Stéphanie Dakpé, Marie-Christine Ho Ba Tho, Tien-Tuan Dao

**Affiliations:** 1 Université de Technologie de Compiègne, CNRS, Biomechanics and Bioengineering, Centre de Recherche Royallieu, Compiègne, CEDEX, CS 60319-60203, France; 2 Department of Maxillo-Facial Surgery, CHU Amiens-Picardie, 80000 Amiens, France; 3 CHIMERE Team, University of Picardie Jules Verne, 80000 Amiens, France; 4 Université de Lille, CNRS, Centrale Lille, UMR 9013—LaMcube—Laboratoire de Mécanique, Multiphysique, Multiéchelle, 59000 Lille, France

**Keywords:** 3D morphable model, 3D pre-trained model, deep learning, fast 3D face reconstruction, Kinect-driven reconstruction, MRI, single image

## Abstract

The 3D reconstruction of an accurate face model is essential for delivering reliable feedback for clinical decision support. Medical imaging and specific depth sensors are accurate but not suitable for an easy-to-use and portable tool. The recent development of deep learning (DL) models opens new challenges for 3D shape reconstruction from a single image. However, the 3D face shape reconstruction of facial palsy patients is still a challenge, and this has not been investigated. The contribution of the present study is to apply these state-of-the-art methods to reconstruct the 3D face shape models of facial palsy patients in natural and mimic postures from one single image. Three different methods (3D Basel Morphable model and two 3D Deep Pre-trained models) were applied to the dataset of two healthy subjects and two facial palsy patients. The reconstructed outcomes were compared to the 3D shapes reconstructed using Kinect-driven and MRI-based information. As a result, the best mean error of the reconstructed face according to the Kinect-driven reconstructed shape is 1.5±1.1 mm. The best error range is 1.9±1.4 mm when compared to the MRI-based shapes. Before using the procedure to reconstruct the 3D faces of patients with facial palsy or other facial disorders, several ideas for increasing the accuracy of the reconstruction can be discussed based on the results. This present study opens new avenues for the fast reconstruction of the 3D face shapes of facial palsy patients from a single image. As perspectives, the best DL method will be implemented into our computer-aided decision support system for facial disorders.

## 1. Introduction

The patients, who are involved in facial palsy or facial transplantation, experience facial dysfunctionalities and abnormal facial motion due to altered facial nerves and facial muscle systems [1,2]. This leads to unwanted facial movements, such as dysfunctionalities of speaking, eating, and the unnatural relaxation of mouth corners drop, eyelid closure, and asymmetrical facial expressions [3,4]. Recently, computer-aided decision systems have been developed to provide objective and quantitative indicators to better diagnose and to optimize the rehabilitation program [5]. The 3D reconstruction of an accurate face model is essential for providing reliable feedback. This is currently achieved by using medical imaging [6] and different sensors, such as Kinect or 3D scanners [7,8]. Thus, this allows for the analysis of the face with external (i.e., face deformation) and internal (i.e., facial muscle mechanics) feedback for the diagnosis and rehabilitation process of facial palsy and facial transplantation patients [9].In the past few decades, facial analysis has attracted great attention due to its numerous exploitations in human-computer interaction [10,11], animation for entertainment [12,13,14], and healthcare systems [15,16]. Facial analysis from images remains a challenge due to variation poses, expressions, and illumination. 3D information can be used in order to cope with these variation problems. 3D facial data can be acquired from medical imaging [17,18], 3D scanners [7,8], stereo-vision systems [19], or RGB-D devices, such as Kinect. The use of medical imaging leads to a very accurate 3D model, but this is not appropriate for an easy-to-use, cheap, and portable system. The use of a depth camera, such as Kinect, can lead to a reasonable accuracy level while keeping the cheap cost, easy-to-use, and portable requirements, but the developed system depends strongly on the selected sensors. In fact, this could alter the future applicability due to stopped production, such as in the case of the Kinect V2 camera. Thus, it is necessary to have a more flexible and open method for building 3D information rather than using specific scanning devices.

Numerous applications have been developed to perform 3D shape reconstruction from 2D images, such as in the computer animation field when creating 3D avatars from images or in entertainment fields (e.g., virtual reality or gaming applications) where it is necessary to embed the user avatar into the system [20]. Chien-Hsu Chen et al. [20] (2015) proposed the use of an augmented reality-based self-facial modeling system. The system overlays 3D animation of participant faces for six basic facial expressions, allowing them to practice emotional assessments and social skills. The virtual avatars’ 3D head and face models were created to suit patients, and then the system was applied for patients to practice emotional and social skills by allowing the virtual avatar models to perform six fundamental facial expressions. Additionally, a reconstructed 3D face provides biometric features for security purposes, such as human identification [21,22] and human expression recognition [23]. In fact, the face of a person can be used as particular biometric evidence, along with other biometric information, such as what a person has (e.g., iris, fingerprint, retina, etc.) or produces (e.g., gait, handwriting, voice, etc.) [21]. Biometric facial recognition is an appealing biometric technique because it relies on the same identifier that people use to differentiate one person from another: the face.

Various methods have been developed to estimate 3D face shapes from one image or multi-views images [24]. Three different approaches have been applied to reconstruct the 3D shape from 2D information. The first approach uses the statistical model fitting with a prior 3D facial model to fit the input images [25,26,27]. The second approach is based on photometric stereo, which is suitable for multiple images, and combines a 3D template face model with photometric stereo methods to compute the surface normal of the face [28,29]. The third approach uses deep learning to learn the shape and appearance of the face by training 2D-3D mapping functions [30,31].

The first approach uses a prior statistical 3D facial model to fit the input images [25,26,27]. In fact, 3D face reconstruction from 2D images is an ill-pose problem. It needs some types of previous knowledge. In order to find the solution, statistical 3D face models are preferred methods for incorporating this previous knowledge since they encode facial geometric variations. The 3D morphable model is a statistical 3D face model built from a set of 3D scans of heads. This model includes both the shape and the texture of the face. There are several existing 3D statistical models from the last few decades. For example, Blanz and Vetter (1999) created a 3DMM in UV space from 200 young adults, including 100 females and 100 males [32]. The well-established Basel Face Model (BFM) [33] was built from 200 subjects (100 males and 100 females with an average age of 24.97 years old, from 8 to 62) with most of the subjects being Caucasian. Several examples are the FaceWarehouse model [34], FLAME model [35], and BFM 2017 model [36]. In particular, the FaceWarehouse model was built by Cao et al. [34] (2014) from depth images of 150 participants. Each has 20 different expressions, and the age range is between 7 and 80 years old. Afterward, by identifying parameters of the linear combination of 3D statistical model bases that best matches the provided 2D image, a new 3D face can be reconstructed from one or more images. For example, Wood, Erroll, et al. [37] fit a morphable model to dense landmarks covering the entire head, including the eyes and teeth to a wild image to reconstruct monocular 3D face. This method is effective for predicting dense landmarks for a real-time system at over 150FPS.

The second approach is based on the photometric stereo. The method is suitable for multiple images. The method combines a 3D template face model with photometric stereo algorithms to compute the surface normal of the face [28,29]. Kemelmacher-Shlizerman and Basri [28] reconstructed the 3D face of a person based on a template model. This method estimated each of the three elements, including the surface normal, albedo, and depth map alternatively by fixing the two remaining. In particular, the spherical harmonic parameters γ were estimated by fixing the albedo and the normal of the template model, while fitting the reference shape into the input image. The depth map from the input image is computed by using pre-computed γ and albedo parameters. Finally, the albedo was recovered using pre-computed γ and the depth map. Without the assumption of uniform surface albedos, a robust optimization approach was developed to accurately calibrate per-pixel illumination and lighting direction [38]. The input images are then semantically segmented using a customized filer along with the geometry proxy to adjust hairy and bare skin areas.

The third approach uses deep learning to learn the shape and appearance of the face by training 2D-3D mapping functions. The method encodes prior knowledge of the 3DMM into the weights of the deep neural network. Several examples can be mentioned. Kim et al. [39], for example, rendered synthetic images using parameters predicted based on the trained neural network from a real image. Then these synthetic images were added to the training set in each iteration. As the result, after each iteration, the training dataset was augmented by combining the data generated by the training network. Li et al. [40] and Pan et al. [41] used encoder-decoder architecture. The encoder part makes the dimensional reduction of the input image to find new representative features, while the decoder part makes use of the new representative features to reconstruct the 3D facial geometry of a person.

Even if these approaches lead to very good accuracy levels for 3D face reconstruction and are able to reconstruct 3D subject-specific face reconstruction, the 3D face shape reconstruction of facial palsy patients is still a challenge, and this has not been investigated. In fact, the reconstruction of patient-specific 3D face models may be useful for assessing the severity degree of facial palsy patients, such as their symmetry. Additionally, merging a 3D face model reconstructed from the patient with an animation of practicing rehabilitation exercises can generate a realistic animation. This may help patients learn facial motion and practice rehabilitation exercises more effectively [42]. The objective of the present study was to apply these state-of-the-art methods to reconstruct the 3D face shape models of facial palsy patients in natural and mimic postures from one single image. Besides, several ideas for increasing the accuracy of the reconstruction can be discussed based on the results. Then, based on the outcomes, the best method will be selected and implemented into our computer-aided decision support system of the facial disorders.

## 2. Materials and Methods

**Figure 1:**
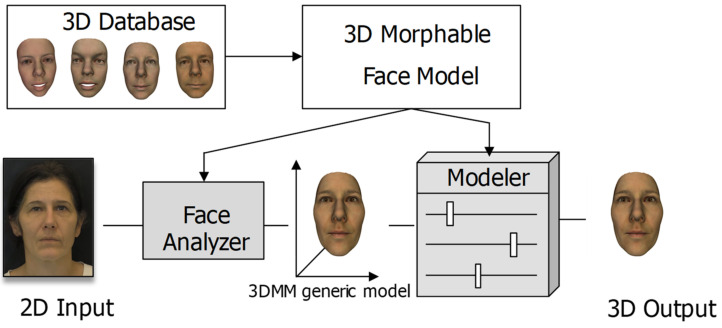
The general framework for reconstructing a 3D face of an individual.

The general framework for reconstructing the 3D face of an individual is illustrated in Figure 1. To begin with, a 3D morphable face model is generated from a set of 3D face scans. Then, from the input 2D image, the learned model extracts features and estimates the corresponding parameters of the 3D morphable model.

### 2.1. Materials

In order to reconstruct the 3D face from a 2D image, we used a dataset of two healthy subjects (one male and one female) and two facial palsy patients (two females) collected from CHU Amiens (France). Each healthy subject or patient signed an informed consent agreement before the data acquisition process. The protocol was approved by the local ethics committee (no2011-A00532-39). The subject performed several trials with neutral positions and facial mimic positions, such as smile, [e], and [u] pronunciation. Our developed Kinect-based computer vision system [5] was used to capture the high density (HD) point clouds of the face as well as the RGB image from Kinect sensors. The images were captured where each subject was positioned in front of the camera with a distance of about 1 m. The RGB image was used for 3D shape reconstruction with the deep learning models. The HD point cloud was used to reconstruct the 3D shape for validation purposes. Moreover, 3D face scans using an MRI were also available for validation purposes.

Two other datasets were collected in order to test more patients with facial palsy. The first dataset of 8 patients was collected in an unconstrained condition [43]. The second dataset of 12 patients was obtained from the Service Chirurgie Maxillo-Faciale CHU Amiens (Prof. Stéphanie DAKPE, Dr. Emilien COLIN) from a pilot study « Etude pilote d’évaluation quantitative de l’attention portée aux visages présentant une paralysie faciale par oculométrie (eye-tracking) » with clinical trial registered (ClinicalTrials.gov Identifier: NCT04886245-Code promoteur CHU Amiens-Picardie: PI2019_843_0089-Numéro ID-RCB: 2019-A02958-49).

### 2.2. Method 1: Fitting a 3D Morphable Model

**Figure 2:**
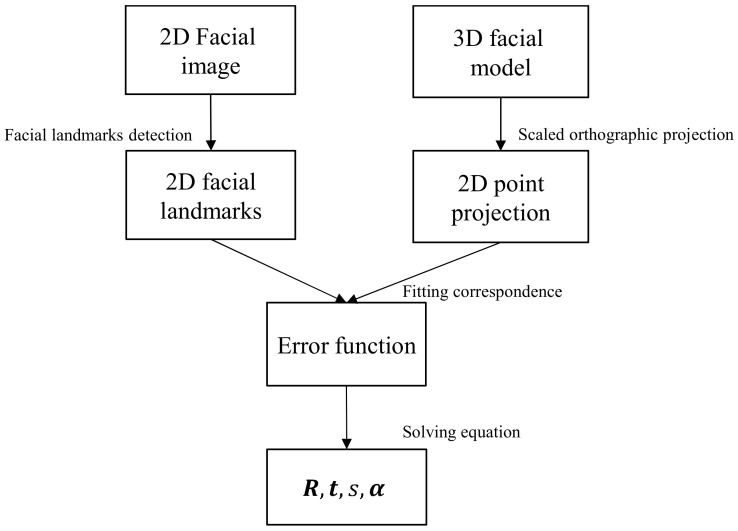
Pipeline to estimate the shape parameters of the 3DMM.

The information processing pipeline of the 3D morphable modeling approach [25] to reconstruct the 3D face shape from a single image is illustrated as in Figure 2. Firstly, a set of 2D facial landmarks are detected from the input image by existing face detectors [44,45]. Secondly, the scaled orthographic projection projects another set of landmarks from the 3D model to obtain 2D points in the image plane corresponding with those points obtained from the 2D image. This step results in an equation that parameterizes the pose and shape parameter. During the next step, a cost function is built to minimize the error between the 2D facial landmarks from the 3D model and 2D facial landmarks from the 2D facial image.

#### 2.2.1. 3D Basel Morphable Model

In the present study, the Basel 3D Morphable model (3DMM) was used [33]. This model was built from a set of 3D faces from a scan of 100 females and 100 males by presenting the face model in terms of trained vector spaces as shape vector spaces. Each face is parameterized in the form of angular meshes with 53,490 vertices. The $S={\left(x_{1},y_{1},z_{1},\ldots ,x_{m},y_{m},z_{m}\right)}^{t}$ is the shape vector for m=53,490 vertices. Each vector is in 53,490×3=106,470 dimensions.

During the next step, all shape vectors of all 200 subjects were concatenated to obtain the matrix of shape S (106,470×200 dimensional matrix). The principal component analysis (PCA) was utilized to decompose the shape matrix resulting in a set of linear combinations of shape bases and texture bases. The PCA is usually a technique for reducing the dimension of the high dimensional data and still remains the largest information source. The constitutive equation of this approach is given in the following equation:

(1) $$S=S_{0}+S_{i}\alpha_{i}$$

where S (106,470×200 dimension matrix) is the shape matrix of 200 subjects and $S_{0}$ (106,470×200 dimensional matrix) is the mean shape matrix with a mean shape vector at each column. $S_{i}$(106,470×106,470 dimensional matrix) is the principal component of the eigenvector of the covariance matrix from the shape matrix, and $\alpha_{i}$ (106,470×200 dimensional matrix) is the eigenvalue, which stands for the coefficients of the shape. The number of the $S_{i}$ column and the $\alpha_{i}$ row can be reduced by choosing the large value of the eigenvalue and dropping out the smaller value of the eigenvalue.

**Figure 3:**
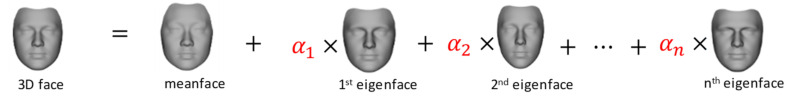
Patient-specific 3D face model was reconstructed using mean face, eigenfaces, and coefficients.

In the PCA decomposition, the mean shape and the shape bases are shared for every specific individual, as presented in Figure 3. This means that the mean shape $S_{0}$ and the shape bases ($S_{i}$) are the same for every subject in the training set; those can be assumed as the population and can be used for other subjects that are different from the subject in the training set. Therefore, from a given facial image, the 3D face can be reconstructed by finding the coefficients of the specific shape.

#### 2.2.2. Model Fitting

##### Facial Landmark Detection

Based on the input image, facial landmarks were detected using the pre-trained facial landmark detector (dlib library) for the iBUG300-W database [44] from “300 Faces In-the-Wild Challenge” for automatic facial landmark detection. The method detects 68 facial landmarks using the Active Orientation Model, which is a variant of an Active Appearance Model [46].

##### Pose from Scaled Orthographic Projection

The rotation matrix and translation vector of the face were used to transform a 3D face from the space coordinate system into the camera system. The scaled orthographic projection assumes that the depths from every point in the face to the camera are not various from one another, therefore, the mean depth of the face can be the same for every point on the face. The projection of the 3D face to the image plane can be estimated using rotation $R\in {\mathbb{R}}^{3\times 3}$, translation $t\in {\mathbb{R}}^{2}$, and the scale factor S∈ℝ. This is expressed in the following equation:

(2) $$SOP\left[f,\;R,t,s\right]=s\left[\begin{array}{ccc} 1 & 0 & 0\\ 0 & 1 & 0 \end{array}\right]Rf+st$$

where $SOP\left[f,\;R,t,s\right]$ are the 2D points of the 3D face in the image plane by scaled orthographic projection; f. represents the 3D facial points.

Additionally, the f in the projection equation can be expressed using the shape equation as follows:

(3) $$f=f_{0}+f_{i}\alpha_{i}$$

where f is several points in the 3D face, $f_{0}$ is the corresponding points of the mean shape face, $f_{i}$ corresponds to the shape bases and $\alpha_{i}$ is the shape coefficients that can be used to reconstruct the 3D face.

##### Fitting Correspondences

Optimizing the difference between 2D facial landmarks detected from the input image and corresponding 2D facial landmarks projected from the 3D model could result in the pose parameter, including rotation, translation, and scale factor, along with the shape parameter (shape coefficients) as follows:

(4) $$E\left[\alpha ,\;R,t,s\right]=\frac{1}{L}s\sum_{i=1}^{L}x_{i}∥-SOP\left[v,\;R,t,s\right]∥$$

This problem can be solved by the iterative algorithm POSIT (POS with iteration) [47]. The solution is able to estimate the rotation R, translation t, and scale factor s of the input face and the shape parameter $\alpha_{i}$ for reconstructing the 3D face of the specific image.

### 2.3. Method 2: 3D FLAME (Faces Learned with an Articulated Model and Expressions) Model

The second method reconstructs the 3D face using the approach of Yao et al. [48], in which the coefficients of the FLAME model [49] were learned from the pre-trained model ResNet50 [50].

#### 2.3.1. The Principle

The geometry shape method used an established 3D statistical head model, namely FLAME [49], which can generate the face with different shapes, expressions, and poses. The model is a linear combination of identity $\beta \in {\mathbb{R}}^{\left|\beta \right|}$, expression $\psi \in {\mathbb{R}}^{\left|\psi \right|}$ with linear blend skin, and pose $\theta \in {\mathbb{R}}^{3k+3}$ (k=4 includes the neck, jaw, and two eyeballs). The FLAME model is defined as follows:

(5) $$M\left(\beta ,\psi ,\theta \;\right)=W\left(T_{P}\left(\beta ,\psi ,\theta \;\right),J\left(\beta \right),\theta ,W\right)$$

(6) $$T_{P}\left(\beta ,\psi ,\theta \;\right)=T+B_{S}\left(\beta ,S\right)+B_{P}\left(\theta ,P\right)+B_{E}\left(\psi ,E\right)$$

where $W\left(T,J,\theta ,W\right)$ is the blend skinning function rotating a set of vertices in $T\in {\mathbb{R}}^{3n}$ around joints $J\in {\mathbb{R}}^{3k}$, which is smoothed by the blend weights $W\in {\mathbb{R}}^{k\times n}$.

The appearance model was converted from the Basel Face Model and generated a UV albedo map $A\left(\alpha \right)\in {\mathbb{R}}^{d\times d\times 3}$, where albedo parameter $\alpha \in {\mathbb{R}}^{\left|\alpha \right|}$.

The camera model aims to project 3D vertices onto the image plane $v=s\Pi \left(M_{t}\right)+t$, where $M_{t}\in {\mathbb{R}}^{3}$ is a vertex in M, $\Pi \in {\mathbb{R}}^{2\times 3}$ is the orthographic projection matrix from 3D to 2D, and S∈ℝ and $t\in {\mathbb{R}}^{2}$ represent the isotropic scale and 2D translation, respectively.

The illumination model finds the shaded face image based on spherical harmonics [51], and texture rendering is based on the geometry parameters $M\left(\beta ,\psi ,\theta \;\right)$, albedo, and camera information.

#### 2.3.2. Model Learning

Reconstructing the face of the patients based on two steps: coarse reconstruction and detail reconstruction.

A coarse reconstruction was performed by training an encoder $E_{c}$ consisting of ResNet50, which minimizes the variation between the input image I and the synthesis image I_r_, which is generated by decoding the latent code of the encoded input image. The latent code contains a total of 236 parameters of the face model, such as geometric information (100 shape parameters of β, 50 expression parameters of ψ, and pose parameters θ), 50 parameters of appearance information α, camera and lighting conditions.

The loss function for the $E_{c}$ network computes the differences between the input image I and the synthesis image I_r_, and consists of the (1) landmark loss ($L_{landmark}$) of 68 2D key points on the face; (2) eye closure loss ($L_{eye}$), penalizing the relative variation between landmarks on the upper and lower eyelid; (3) photometric loss ($L_{photometric}$), comparing between input image I and the synthesis image I_r_; (4) identity loss ($L_{identity}$), computing the cosine similarity which presents the fundamental properties of the patient’s identity; (5) shape consistency loss ($L_{shape}$), computing the differences between the shape parameters (β) from different images of the same patient; and (6) regularization ($L_{regularization}$) for shape, expression, and albedo as follows:

(7) $$L_{coarse}=L_{landmark}+L_{eye}+L_{photometric}+L_{identity}+L_{shape}+L_{regularization}$$

Then, the detail reconstruction assists in augmenting the coarse reconstruction with the different details, such as wrinkles and facial expressions, using a detailed UV displacement map. The detail reconstruction trains an encoder $E_{d}$, which is the same architecture $E_{c}$ to output 128 latent codes δ relating to the patient-specific details. The loss function for the $E_{d}$ network contains (1) photometric detail loss ($L_{photometric\;detail}$) based on a detail displacement map, (2) implicit diversified Markov random field loss ($L_{mrf}$) [52] related to geometric details, (3) soft symmetry loss ($L_{symmetry}$) to cope with self-occlusions of face parts, and (4) detail regularization ($L_{regularization\;detail}$) to reduce noise as follows:

(8) $$L_{dEtail}=L_{photometric\;detail}+L_{mrf}+L_{symmetry}+L_{regularization\;detail}$$

### 2.4. Method 3: Deep 3D Face Reconstruction

The third method relates to the deep 3D face reconstruction approach of Yu et al. [53], in which the coefficients of the 3D morphable model of the face were learned from the pre-trained model ResNet50 [50].

#### 2.4.1. 3D Morphable Model

The shape S and the texture T of the 3DMM were presented as follows:

(9) $$S=S\left(\alpha ,\beta \right)=\bar{S}+B_{id}\alpha +B_{exp}\beta$$

(10) $$T=T\left(\delta \right)=\bar{T}+B_{t}\delta$$

where $\bar{S}$ and $\bar{T}$ are the mean shape and texture of the face model; $B_{id}$, $B_{exp}$, and $B_{t}$ are the principal component vectors based PCA presenting for identity, expression, and texture; and respective coefficients vectors are α,β, and δ.

The scene illumination was modeled using spherical harmonics coefficients $\gamma_{b}\in {\mathbb{R}}^{9}$. The radiosity of a vertex $S_{i}$ was computed as $C\left(n_{i},t_{i}\right)=t_{i}\cdot \overset{B^{2}}{\underset{b=1}{\sum }}\gamma_{b}\Phi_{b}\left(n_{i}\right)$, where $n_{i}$ and $t_{i}$ are the surface normal and skin texture of the vertex $s_{i}$, and $\Phi_{b}$ is the spherical harmonics basis function.

The pose p of the face is represented by rotation **R** and translation **t**. All of the unknown parameters (e.g., $x=\left(\alpha ,\beta ,\;\delta ,\gamma ,p\right)\in {\mathbb{R}}^{239}$) are the output of the modified RestNet-50, with the last layer including 239 neurons.

#### 2.4.2. Model Learning

**Figure 4:**
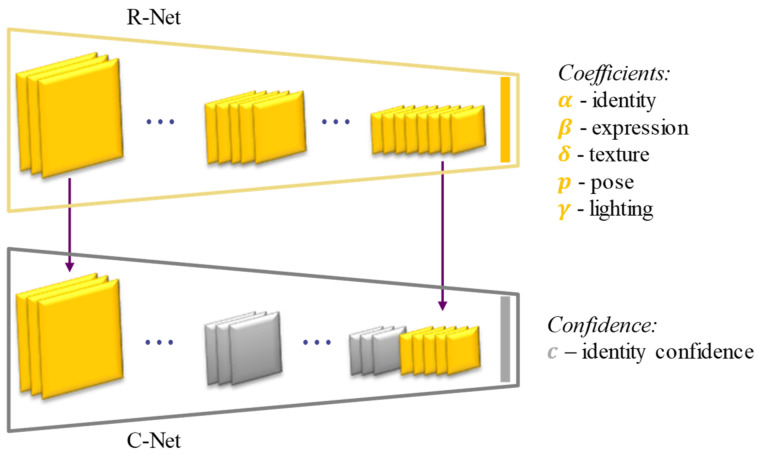
The network architecture for learning the parameters of the face model. The output of models, including coefficients that represent identity (α), expression (β), texture (δ), pose (p), lighting (γ), and identity confidence (c).

The coefficients are the output of the ResNet-50 model, as illustrated in Figure 4, which is modified based on the last fully collected layer and was trained by estimating a hybrid-level loss of image-level loss and perception-level loss, instead of using ground truth labels.

Image-level losses integrate photometric loss for each pixel and landmark loss for sparse 2D landmarks detected from the input image. The photometric loss between the raw (I) and the reconstructed ($I^{\prime }$) images is defined as follows:

(11) $$L_{photo}\left(x\right)=\frac{\sum_{i\in M}A_{i}\cdot {∥I_{i}-I_{i}^{\prime }\left(x\right)∥}_{2}}{\sum_{i\in M}A_{i}}$$

where, with each pixel index i, M denotes the re-projected face region, A is skin color, and ${∥\cdot ∥}_{2}$ is the $l_{2}$ norm.

Landmark loss is computed on 68 landmarks $\left\{q_{n}\right\}$ detected from an input image [54] and landmarks projected of the reconstructed shape onto the image $\left\{q_{n}^{\prime }\right\}$ as follows:

(12) $$L_{lan}\left(x\right)=\frac{1}{N}\sum_{n=1}^{N}\omega_{n}{∥q_{n}-q_{n}^{\prime })∥}_{2}$$

where $\omega_{n}$ is the landmark weight and is set to 20 for the mouth and nose points, while it is set to 0 for others.

Perception-level loss tackles the local minimum issue for CNN-based reconstruction by extracting deep features from the images of the pre-trained FaceNet model for deep face recognition [55] and uses it to estimate perception loss.

(13) $$L_{per}\left(x\right)=1-\frac{\langle f\left(I\right),f\left(I^{\prime }\left(x\right)\right)\rangle }{∥f\left(I\right)∥\cdot f(I^{\prime }\left(x\right)∥}$$

where $f\left(\cdot \right)$ represents the deep feature and 〈·, ·〉 is the vector inner product.

Two regularization losses involving coefficients and textures are added to avoid shape and texture degeneration. The coefficients loss invokes the distribution close to the mean face:

(14) $$L_{coef}\left(x\right)=\omega_{\alpha }∥\alpha ){∥}^{2}+\omega_{\beta }∥\beta ){∥}^{2}+\omega_{\gamma }∥\delta ){∥}^{2}$$

The weights are set to $\omega_{\alpha }=1.0$, $\omega_{\beta }=0.8$, and $\omega_{\gamma }=0.0017$. The texture loss is computed by flattening constrain

(15) $$L_{tex}\left(x\right)=\sum_{c\in \left\{r,g,b\right\}}var\left(T_{c,R\left(x\right)}\right)$$

where R is a pre-defined region of the skin at the cheek, nose, and forehead.

### 2.5. Validation versus Kinect-Driven and MRI-Based Reconstructions

**Figure 5:**
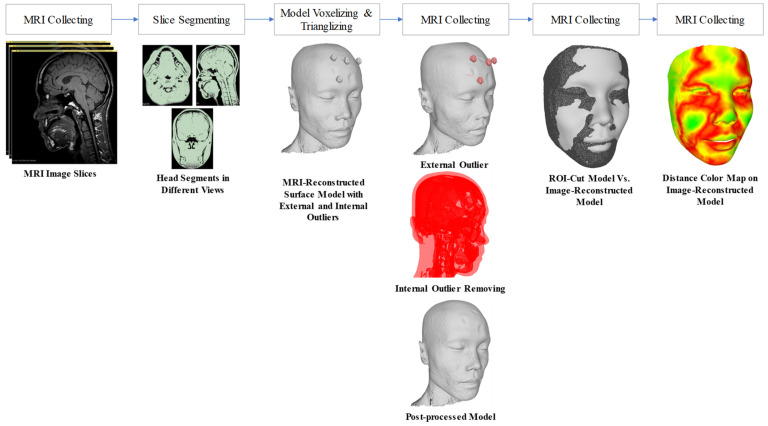
Reconstructed 3D face shape from the MRI images and segmentation. The 3D face shape was finally registered to the coordinate system of the image-based reconstructed face model before calculating the Hausdorff distances.

The reconstructed outcomes from the above three methods were compared to the 3D shape reconstructed from the Kinect-driven and MRI-based shapes. The 3D Kinect-driven shape was reconstructed by using our computer vision system; please refer to the [56] for detailed information on the processing method. Specifically, the MRI (magnetic resonance imaging) images were segmented using the semi-automatic method with the 3D Slicer software, as shown in the Figure 5. 3D shapes were saved in the STL format for further comparison. The Hausdorff distance [57] was used to estimate the error of the reconstructed face compared with ground truth data from MRI and Kinect devices. The CPU with configuration core i9-9800H CPU, 2.30 GHz, 32.0 GB RAM, and 16GB NVIDIA Quadro RTX 5000 GPU was used to predict 3D faces.

## 3. Computational Results

**Figure 6:**
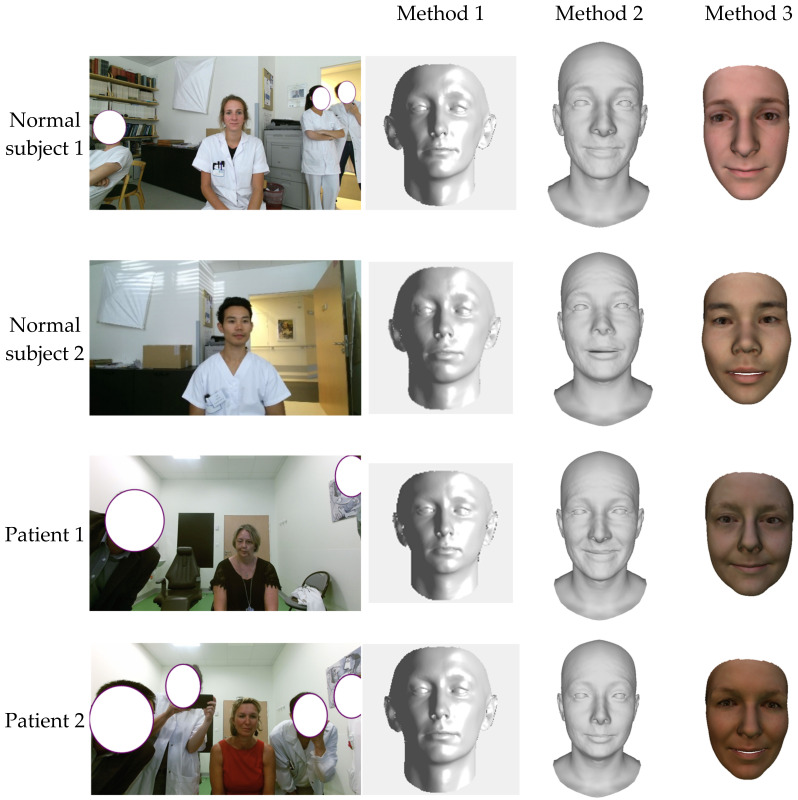
3D face reconstruction from an input image.

The input images of the frontal face of the two facial palsy patients and two healthy subjects are used to reconstruct the corresponding patient-specific face. The reconstructed 3D face shapes are shown in Figure 6 with three applied methods. Comparing the three methods, the second method can reconstruct wrinkles with a full head instead of the cropped face, compared with methods one and three. The second and third methods were able to reconstruct the shape detail parameters, such as shape, pose, and expression, while the first method only reconstruct the subject in the neutral position.

**Figure 7:**
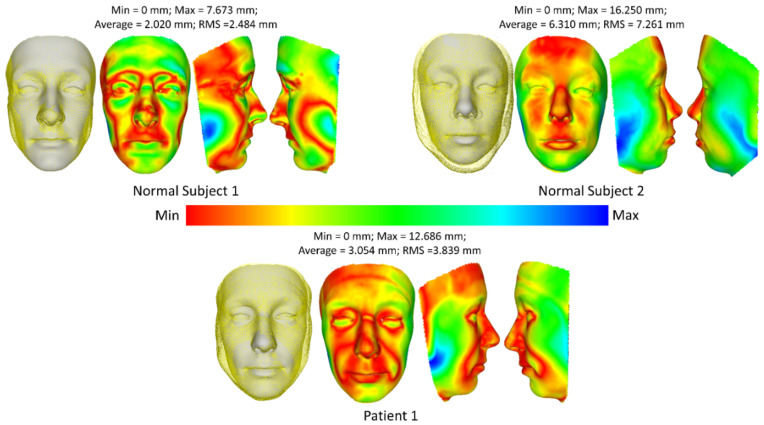
Comparison of 3D face reconstruction (grey) and 3D face reconstruction from MRI (yellow) using the first method (fitting a 3DMM).

The performance was then quantified by comparing it with the 3D face obtained from the 3D camera Kinect and the MRI image. The 3D face from the MRI-based method can be treated as the ground-truth data of the person, while the 3D face from the Kinect-based method reconstructs the face with an error of about 1mm. Figure 7 demonstrates the smallest error of the 3D face reconstructed from the input image and the 3D face from the MRI-based method for the first method (fitting a 3DMM). Only three subjects (two normal subjects and one patient) were estimated because the MRI data of the second patient is not available. The average error of the three subjects is from 2.020 mm to 6.310 mm. The smallest error is observed in the center of the face area, while the performance suffers heavily at the jaw. This is because the input image is in the frontal area of the face, while the jaw part is occluded from the frontal face image.

**Figure 8:**
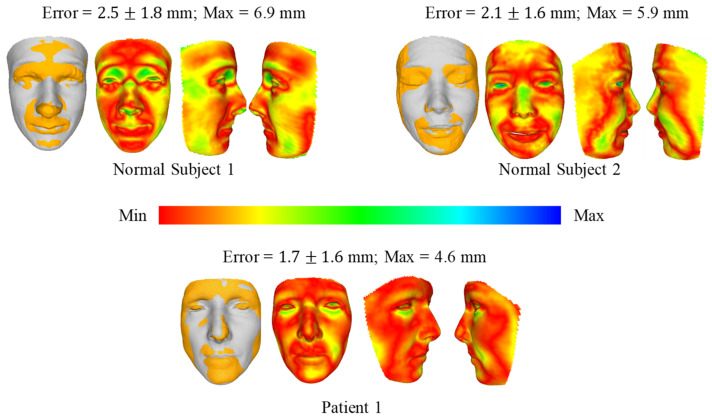
Comparison of 3D face reconstruction (grey) and 3D face reconstruction from MRI (yellow) using the second method (DECA).

**Figure 9:**
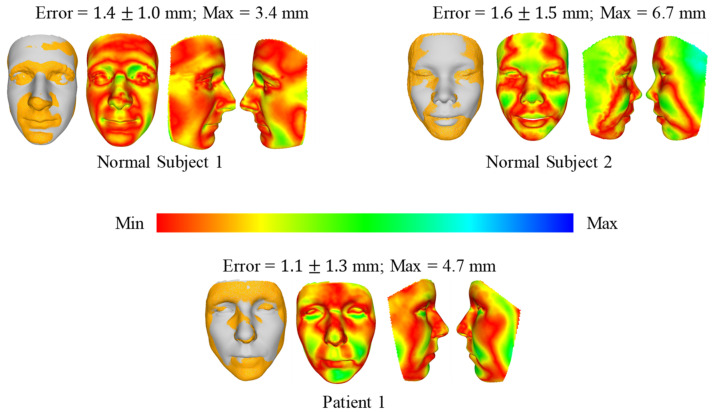
Comparison of 3D face reconstruction (grey) and 3D face reconstruction from MRI (yellow) using the third method (deep 3D face reconstruction).

Figure 8 and Figure 9 show the comparison of 3D face reconstruction (grey) and 3D face reconstruction from MRI (yellow) using the second and third methods, respectively. The average error of the three subjects is from 1.7 mm to 2.5 mm. These errors for the third method range from 1.1 mm to 1.6 mm.

**Figure 10:**
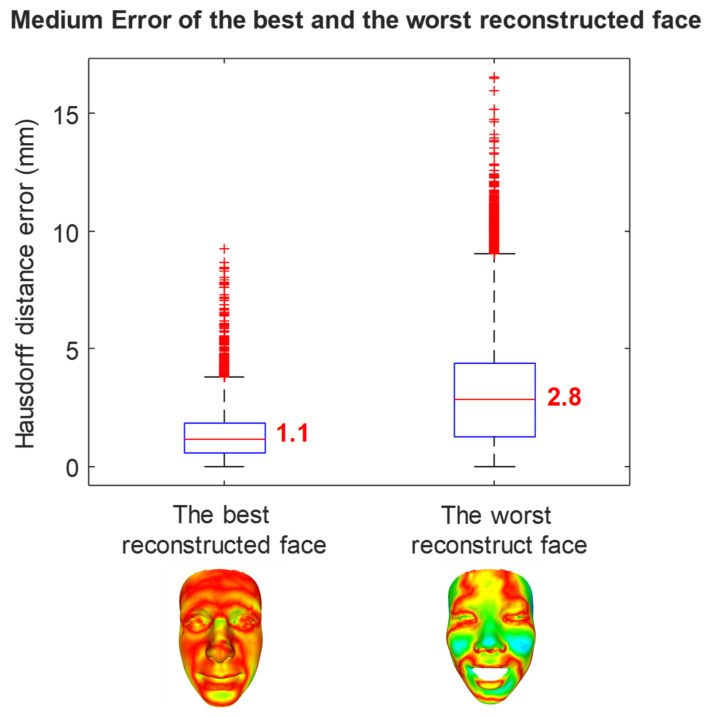
The error of the best and the worst prediction cases of the third method compared with MRI ground truth data.

The best prediction of the third method compared with the MRI ground truth data is 1.1 mm with a maximum error of 3.7 mm, while the worse prediction is 2.8 mm with a maximum error of 9.1mm, as shown in Figure 10.

All comparison mean error ranges are reported in Table 1 for all subjects and patients in the neutral position. The mean error of all subjects from the third method is smaller than that in the second and the first methods.

**Figure 11:**
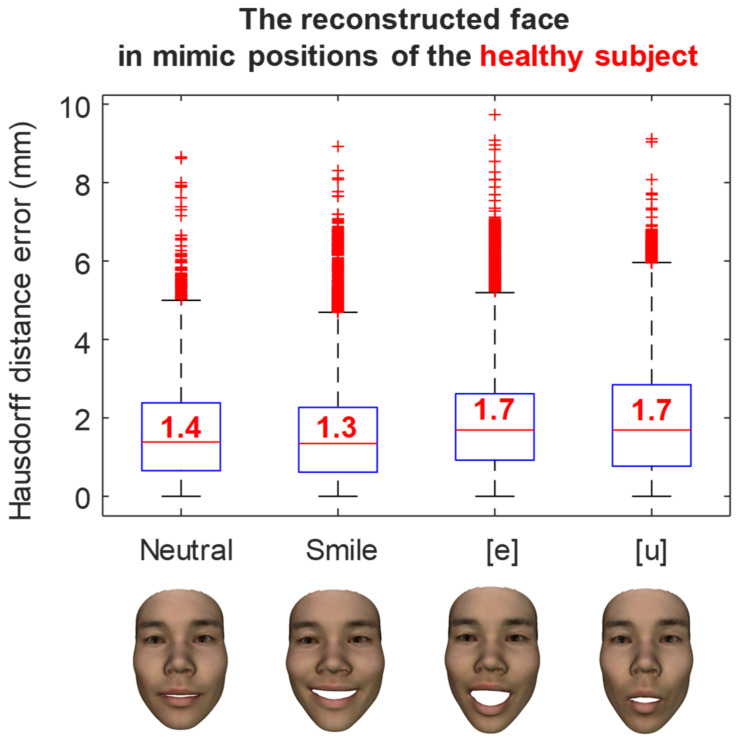
The error of the reconstructed face in mimic position of a healthy subject.

**Figure 12:**
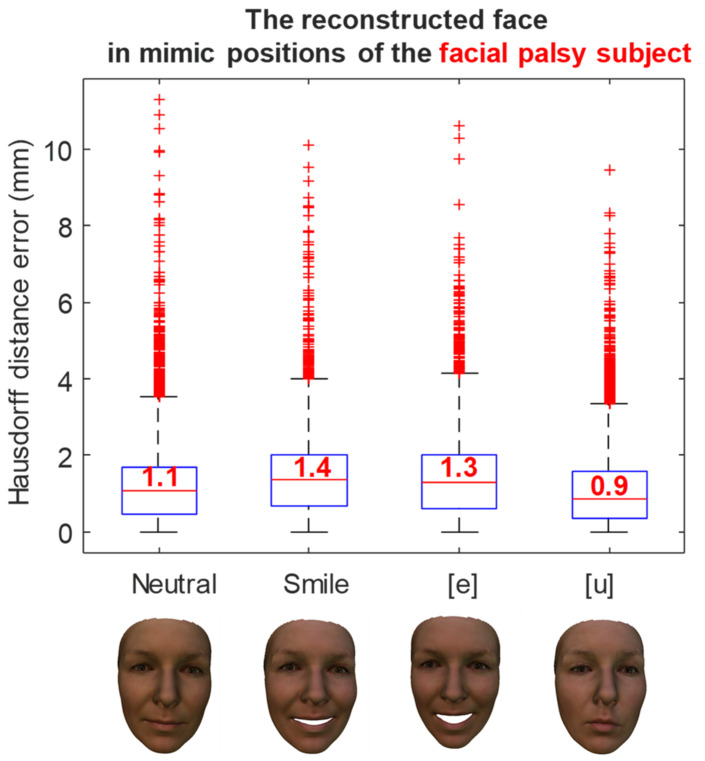
The error of the reconstructed face in mimic position of a facial palsy subject.

The reconstruction errors of a healthy subject in various mimic positions are shown in Figure 11. In the neutral position, the medium reconstruction error is 1.4 mm, while this error is 1.3 mm and 1.7 mm in smile, and [e] and [u] pronunciations, respectively. Similarly, the reconstruction errors of a facial palsy patient in various mimic positions were shown in Figure 12. The medium reconstruction error is 1.1 mm, 1.4 mm, 1.3 mm, and 0.9 mm in neutral, smile, and [e] and [u] pronunciations, respectively.

**Figure 13:**
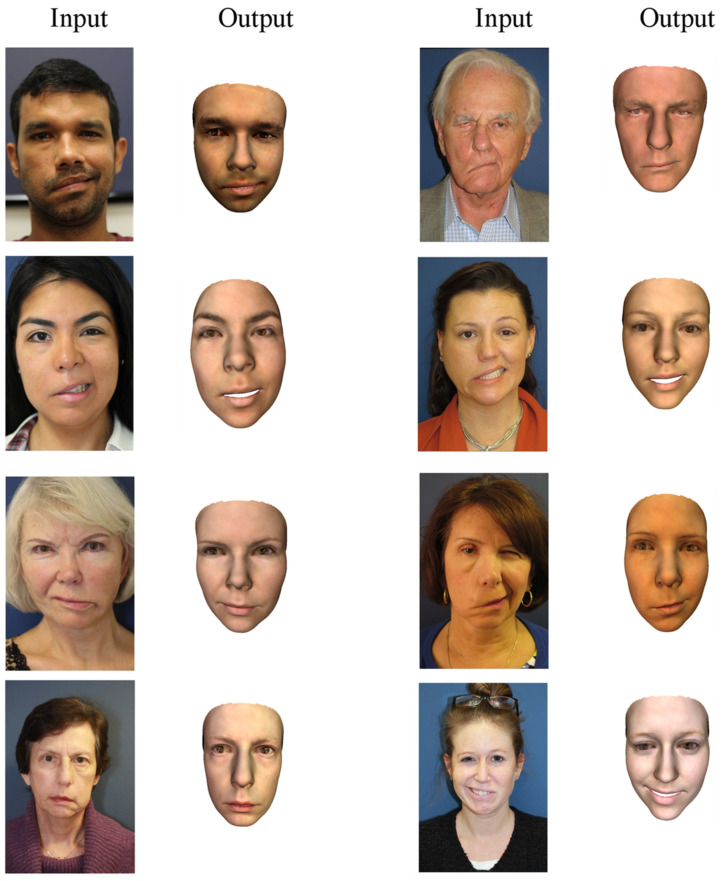
The 3D face reconstruction of facial palsy patients using method 3 (deep 3D face reconstruction) using collected images in open access dataset.

Several examples of 3D face reconstruction were illustrated in Figure 13 using method 3 (deep 3D face reconstruction) for facial palsy patients from 2D images collected in open access.

**Figure 14:**
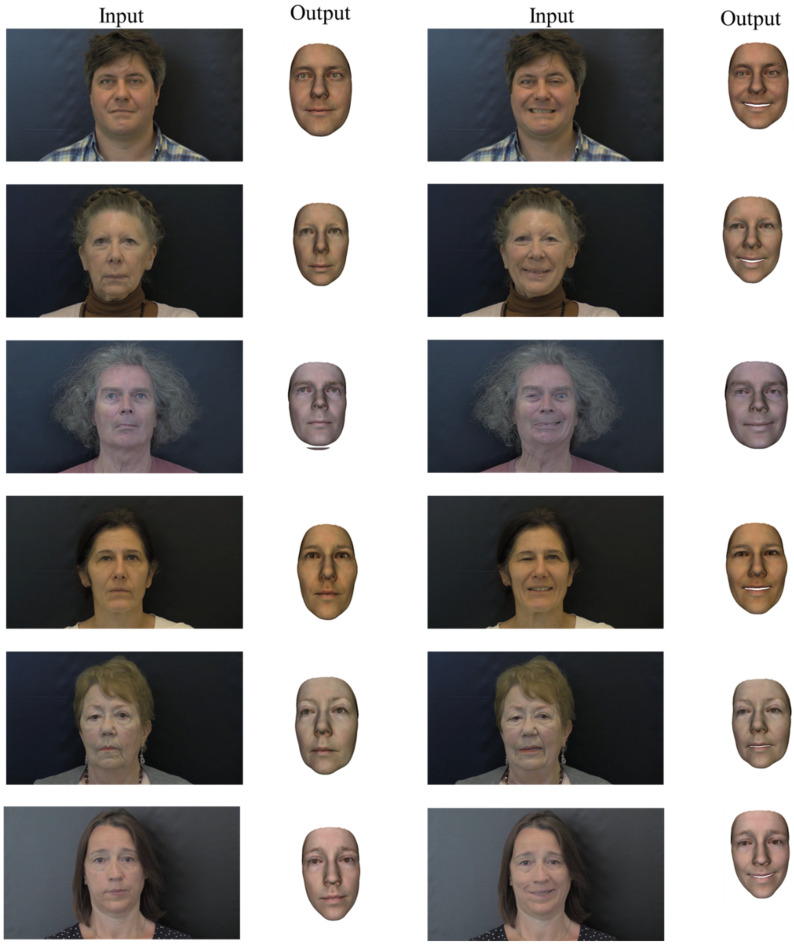
The 3D face reconstruction of the last six facial palsy patients using method 3 (deep 3D face reconstruction) using images from CHU Amiens.

The reconstructed faces of 12 patients in neutral and smiling poses from 2D images obtained at CHU Amiens were illustrated in Figure 14.

For all patients with their face in a neutral position (Figure 13 and Figure 14), the output reconstructed 3D face has quite a close appearance to the individual in the input 2D image. The asymmetric feature of the mouth of all patients can be observed in the reconstructed 3D faces. In the eye region, this asymmetric feature seems less noticeable. In the patients of the second dataset, the asymmetry is not much observed for both positions including neutral and smiling. This is probably due to the degree of severity of the facial palsy, which seems to be less important than the first dataset. This demonstrates the limitation of using the database with normal faces instead of facial palsy.

## 4. Discussion

Fast reconstruction of the 3D face shape plays an important role in the suitable use of computer-aided decision support systems for facial disorders. This allows us to track the normal and abnormal facial deformations in static and dynamic postures, leading to the improved diagnosis and rehabilitation of the involved patients [9,58]. The facial analysis for diagnosis and treatment has mainly been based on 2D images [59,60,61], which remains a challenge due to variation poses, expressions, and illumination. However, the 3D information collected from scanners and other stereo devices is time-consuming and expensive [7,49,62]. Recently, effective data science and deep learning methods have been developed for reconstructing 3D face information from a single image or from multiple images [24]. This opens new avenues for the 3D face shape reconstruction for facial palsy patients. In the present study, we applied three state-of-the-art methods (a morphable model and two pre-trained deep learning models) to reconstruct the 3D face shape in the neutral and facial mimics postures from a single image. Obtained results showed a very good reconstruction error level by using a well pre-trained deep learning model applied for healthy subjects as well as facial palsy patients. The reconstruction is very fast, and this solution is very suitable to be included into a computer-aided decision support tool.

Regarding the comparison with ground truth data from Kinect depth sensor and MRI data, the best mean errors range from 1.5 to 1.9 mm for static and facial mimic postures. These findings are in agreement with the accuracy level reported in the literature. An accuracy comparison for heathy subjects revealed that, in the neutral position, the error range is less than 2mm when comparing the Basel Face Model (BFM), FaceWarehouse model, and FLAME model [49]. Moreover, the error ranges from 5 to 10mm for positions with a large movement amplitude (mouth opening, facial expressions) [49]. In particular, all three methods estimate the 3D face model parameters without any paired ground truth data requirement. For the near frontal view image, all of the methods can reconstruct the 3D face of the patient well in the central face area; however, the first method turns out badly when attempting to keep a low error at the jaw where it is occluded, while two other methods can handle the occlude part in a relatively stable way. This is because both the second and third methods were trained with the loss function associated with the pose change. Interestingly, the first method reconstructs the second healthy subject, who is Asian, with a large error (~6.3 mm), while these errors are relatively smaller (2–3 mm) for other subjects, who are French. The reason for this is that the first method is based on a 3DMM model which was built from mostly Caucasian subjects. While the second and third methods, which were based on the 3DMM model, were trained based on subjects with more diversity in ethnicity, there is not much of a difference in error between each subject when reconstructing the 3D face of all subjects (1.7–2.9 mm and 1.6–2.3 mm for the second and third methods respectively). This might prove that with more diversity in ethnicity when building the 3DMM model, the result of the reconstruction can be better. Method 3 was also applied to reconstruct 3D faces of facial palsy patients from unconstrained conditions (images were captured by any devices) since it has the lowest reconstruction error. The method is good at capturing asymmetric features in the mouth area, but less so in the eyes. This is due to the method’s usage of the FLAME model, which includes various expressions but does not include any patients with facial palsy.

In the present study, the first method fits a 3DMM to a single image based on a scale orthographic projection [25]. The method first detects facial landmarks detected on the input image, then projects the set of corresponding 3D points from the 3D model to obtain 2D points, and finally estimates the shape and pose parameters of the face model by minimizing the error between the 2D facial landmarks from the 3D model and 2D facial landmarks from the 2D facial image. The second used method reconstructs the 3D face based on an established FLAME head model [48]. The method is based on a resNet-50 deep learning model to learn the shape parameters, such as shape, expression, pose, and detail, and appearance parameters, such as albedo and lighting. The model is trained by minimizing the loss function estimated from the input image and the synthesized image generated by decoding the latent code of the encoded input image. The third applied method reconstructs the 3D face of the patient with weakly-supervised learning to regress the shape and texture coefficients from a given input image [53]. This method was also based on a hybrid-level loss function to train the resNet-50 deep learning model.

Regarding the 3D shape reconstruction, our findings confirmed the high accuracy level of the 3D pre-trained deep learning models for facial palsy patients. In particular, the third method was able to reconstruct the geometric details, such as shape, pose, and expression, while the second method reconstructs the face with the wrinkle detail. The findings also revealed that the morphable model provided a lower accuracy level. The second and third methods result in better accuracy due to integrating the loss of both geometric information (e.g., the landmark loss) and appearance information (e.g., the photometric loss), while the first method only counts the landmark loss and ignores the appearance information. Another reason for being lower accuracy is that the first method estimates the shape parameters from the Basel 3D Morphable model [33]. This was only modeled based on the face database of mostly Caucasian subjects with neutral expressions. The second and third methods were based on the 3D face models with a higher diversity in ethnicity and variations in facial expressions, such as the FLAME model [49] and FaceWarehouse [34], respectively. The FLAME model was trained from sequences of 3D face scans that can generalize well to the novel facial data of the different subjects, which is more reliable and flexible for capturing patient-specific facial shapes.

One important limitation of the present study deals with a small number of subjects and patients used for prediction. Another limitation deals with the lack of facial palsy patients in the learning database. This results in the reduction of several facial palsy patients’ features (e.g., asymmetric face, dropping mouth corner, cheek) while reconstructing their 3D face. Thus, a larger and diverse 3D facial database, including facial palsy subjects, should be acquired to confirm our findings and contribute toward a potential clinical application. Moreover, another limitation of the study relates to the usage of the 3D statistical facial model. The first method used a 3DMM which was based on the PCA basis vectors so that the reconstruction of more detailed information, such as expression and wrinkles, can become a hard task. The second and third methods improve that by building a more diverse model with subtler information, such as expression and wrinkles, but still use a linear model which could generate more error due to facial shape variations, which cannot be modeled perfectly using a combination of linear components, as noted in [24,63,64]. Improving the existing 3D face models can be a potential suggestion for future works. Furthermore, the reconstruction result has not been statistically analyzed for each region of the face. This could tackle the uncertainty of the predicted models. Another limitation relates to the effect of the variation of the 2D input images, such as the pose and lighting conditions, which have not been investigated. A variation, along with the larger quantity, of facial palsy patients are needed for improving the result of the reconstruction and should be performed in future work.

## 5. Conclusions

The 3D reconstruction of an accurate face model is essential for providing reliable feedback for clinical decision support. Medical imaging and specific depth sensors are accurate but not suitable for an easy-to-use and portable tool. The recent development in deep learning (DL) models opens new challenges for 3D shape reconstruction from a single image. However, the 3D face shape reconstruction of facial palsy patients is still a challenge, and this has not been investigated.

In this present study, the 3D face shape was reconstructed from a single image for facial palsy patients. The methodology could be used for a single 2D image from any device for reconstructing the 3D face of patients with facial palsy. The methodology used several methods to reconstruct the 3D face shape models of the facial palsy patients in natural and mimic postures from one single image. Three different methods (3D Basel Morphable model and two 3D Deep Pre-trained models) were applied to the dataset of two healthy subjects and two facial palsy patients. Reconstructed outcomes showed a good accuracy level compared to the 3D shapes reconstructed using Kinect-driven reconstructed shapes (1.5±1.1 mm) and MRI-based shapes (1.9±1.4 mm).

This present study opens new avenues for the fast reconstruction of the 3D face shapes of facial palsy patients from a single image. As perspectives, reconstructed faces could be used for the further analysis of the face in terms of expression and symmetry. Furthermore, the best DL method will be implemented into our computer-aided decision support system for facial disorders. 

## Figures and Tables

**Table 1:** Reported error ranges of 3D faces reconstructed using the methodology compared to the 3D faces reconstructed by Kinect and MRI techniques for the validation study.

Method	Subject	Error (mm)	Method	Subject	Error (mm)
Fitting—Kinect comparison	1	2.3±2.9	Fitting—MRI comparison	1	2.0±2.5
	2	6.3±7.6		**2**	6.3±7.3
	3	2.4±2.9		3	3.1±3.8
	**Mean**	3.7±4.5		**Mean**	3.8±4.5
Deca—Kinect comparison	1	2.6±1.9	Deca—MRI comparison	1	2.9±2.1
	2	1.5±1.5		2	2.6±2.1
	3	1.5±1.4		3	2.2±2.0
	4	2.2±1.7		4	1.7±1.6
	**Mean**	1.8±1.6		**Mean**	2.3±1.9
Deep3Dface—Kinect comparison	1	1.7±1.3	Deep3Dface—MRI comparison	**1**	1.6±1.1
	2	1.8±1.3		**2**	2.3±1.6
	3	1.3±1.0		**3**	1.8±1.4
	4	1.4±1.0		**4**	1.8±1.5
	**Mean**	1.5±1.1		**Mean**	1.9±1.4

## Data Availability

No new data were created or analyzed in this study. Data sharing is not applicable to this article.

## List of Abbreviations

2D	Two-dimension
3D	Three-dimension
3DMM	3D morphable model
BFM	Basel face model
CNNs	Convolutional neural networks
DECA	Detailed expression capture and animation
DL	Deep learning
FLAME	Faces learned with an articulated model and expressions
FPS	Frame per second
GAN	Generative adversarial network
GPU	Graphics processing unit
HD	High definition
PCA	Principal component analysis
ResNet	Residual neural network
RGB	Red green blue
RGB-D	Red green blue-depth
SCAI	Sorbonne center for artificial intelligence
SOP	Scaled orthographic projection
